# Do dreams really guard sleep? Evidence for and against Freud's theory of the basic function of dreaming

**DOI:** 10.3389/fpsyg.2013.00017

**Published:** 2013-01-30

**Authors:** Fabian Guénolé, Geoffrey Marcaggi, Jean-Marc Baleyte

**Affiliations:** ^1^Department of Child and Adolescent Psychiatry, Caen University HospitalCaen, France; ^2^National Institute for Health and Medical Research (INSERM), UMR 1077, Caen University HospitalCaen, France; ^3^Department of Psychiatry 95G03, Montéran HospitalSaint-Claude, France; ^4^Faculty of Medicine, University of CaenCaen, France

## Introduction

“*A dream is invariably an attempt to get rid of a disturbance of sleep by means of a wish-fullfilment, so that the dream is a guardian of sleep*”.

Sigmund Freud (1940/1953)

According to the classic theory framed by Sigmund Freud, the basic and teleological function of dreaming is to protect sleep from disruption (Freud, 1900/1953); a quite rational hypothesis since sleep constitutes a vital need for living species (Kryger et al., 2011). This aspect of Freud's dream model—which as a whole is considered as the initial cornerstone of psychoanalysis (Laplanche and Pontalis, 1988)—leads to two empirically testable conjectures, thus allowing its scientific examination: (1) arousal during sleep triggers dreaming; and (2) non-dreaming causes sleep disruption. We review here the experimental, medical, and neuropsychological data which allow testing these two conjectures; in this paper, the term “significant” denotes statistical significance at the *p* < 0.05 level.

## Conjecture #1: arousal during sleep triggers dreaming

A series of studies investigating dream reports after nighttime awakenings with or without prior arousal during sleep allows testing this conjecture. In the first one (Conduit et al., 1997), young adult subjects were awakened after simultaneous light and tone stimulation just below the waking threshold, and mentation reports were compared to those elicited from control awakenings without stimulation. Results showed significantly more dream reports after sleep stimulation than without (83% vs. 40%). More precisely, a significant increase of dream reports under stimulation condition was found for non-rapid eye movement (NREM) sleep stage 2 (72% vs. 17%), but not for rapid eye movement (REM) sleep (100% vs. 84%)^1^. The NREM sleep finding was replicated with tone stimulation (46% vs. 9%; Fedyszyn and Conduit, 2007); by contrast, arousal during REM sleep was not associated with increased dreaming in subsequent studies (Fedyszyn and Conduit, 2007; Stuart and Conduit, 2009). It thus can be concluded that arousal during NREM sleep, not REM sleep, is associated with increased dreaming, which is a major argument supporting conjecture #1 (regarding NREM sleep only).

Arousal during sleep can also be studied in medical conditions entailing destabilization of sleep brain activity by systemic or neuro-peripheral factors. In obstructive sleep apnea syndrome, in which nighttime hypoxemia induces brain arousal, dream recall frequency (DRF) displayed no clear increase in retrospective questionnaire studies (see Schredl, 2009), nor in sleep laboratory studies using nighttime awakenings during REM sleep (Gross and Lavie, 1994; Carrasco et al., 2006). In patients with restless legs syndrome (Schredl, 2001), which was associated in more than 75% of cases with periodic limb movements with arousals during sleep (Michael Schredl, pers. communication), no increase in questionnaire-measured DRF was shown compared to healthy controls. This set of studies, however, entail considerable limits for our perspective, which are that: (1) dream questionnaires only partially reflect nighttime dreaming as a whole (Schredl, 2007); (2) no dream study in the sleep laboratory has been conducted to date in sleep movement disorders, and such studies in obstructive sleep apnea syndrome did not investigate NREM sleep dreaming (Gross and Lavie, 1994; Carrasco et al., 2006); and (3) caution must be taken when drawing general psychophysiological conclusions from pathology. Taking these limits into account, observations made in sleep-arousing medical conditions represent a minor argument against conjecture #1.

According to the Freudian model, another and major source of arousal during sleep is unsatisfied drive demands (Freud, 1917/1953): those which are repressed at wake overtake the sleeping ego's capacity for repression, and thus find a way to consciousness using the primary process^2^. Since this pressure deriving from unconscious contents is thought to be typically increased in neurotic patients (Fenichel, 1946), the psychoanalytic theory predicts that these would display increased dreaming (Grünbaum, 1993). In two questionnaire studies (Schredl et al., 2001; Kuelz et al., 2010), DRF was significantly higher, compared to healthy controls, in patients with panic disorder and in psychotherapeutically-untreated patients with obsessive-compulsive disorder, respectively. Here again, several limits must be mentioned: (1) most patients were under medication, which effects on sleep mentation could eventually be incriminated^3^; (2) sleep discontinuity, which is associated with increased DRF (Schredl, 2007) and a frequent condition in mental disorders, was not controlled; and (3) the same limits of questionnaire-measured DRF as mentioned in the preceding paragraph, regarding their limited reflection of nighttime dreaming as a whole, apply to these studies. Taking these limits into account, observations made in neurotic patients represent a minor argument supporting conjecture #1.

## Conjecture #2: non-dreaming causes sleep disruption

The first set of data allowing testing this second conjecture are those collected in subjects with primary anoneira, i.e., constitutional inability to dream. Indeed, since dreaming is thought to be essential for sleep maintenance, it is expected that primary anoneira will be accompanied by functional problems in sleep initiation and continuity, which would enter the category of primary insomnia (Kryger et al., 2011). Actually, primary anoneira is not known to be commonly associated with primary insomnia (Kryger et al., 2011), which—supposing that conjecture #2 is right—could reflect just its actual rarity. Foulkes reported the cases of two brothers aged 11 and 12 years, who both displayed a specific developmental deficit in mental imagery persistently associated with an extremely low REM dream recall, and mentioned no sleep problems (Foulkes, 1999). Pagel studied a group of adults with primary anoneira, whose absence of dreaming had been confirmed through REM and NREM awakenings, and reported no increase in sleep problems (Pagel, 2003). However, the study was conducted within the consulting population of a sleep medical center, with most of patients suffering from sleep obstructive apnea, and the comparison was made only between non- and rare-dreamers. Taking their limits into account, data collected in primary anoneiric subjects represent a minor argument against conjecture #2.

Conjecture #2 can also be investigated in secondary anoneiric individuals, i.e., in patients with global cessation of dreaming following brain damage. Solms performed a vast clinico-anatomical study in this domain, which identified two lesion sites determining cases of secondary anoneira: (1) bilateral damage to the ventromesial quadrant of the frontal lobe; and (2) uni- or bilateral damage to the occipito-temporo-parietal junction (Solms, 1997). Regarding sleep disruption, results showed that these brain-damaged patients with secondary anoneira manifested significantly more sleep disruption than brain-damaged patients (equivalent size) without anoneira (42% vs. 32%). It is worth noting however that, according to the neuro-psychoanalytical model set by Panksepp and Solms (2012), only patients with posterior lesions would be expected to exhibit an anoneira-induced insomnia. Indeed, whereas patients with uni- or bilateral damage to the occipito-temporo-parietal junction are thought to display a failure of dream's psychic figurability (Botella and Botella, 2005), originating in a cognitive deficit in mental imagery processes (see Solms, 2000), patients with bilateral damage to the ventromesial quadrant of the frontal lobe, whose brain damage impairs the emotional SEEKING system (Panksepp, 1998), are thought to loose a great part of their dreaming activity by being deprived of drive demands^4^ (Solms, 2000). Thus, since patients belonging to this second category have no reason to be sleep-disturbed according to the model, their inclusion in the comparison could be considered as a confusion bias, and could have limited the contrast between groups. Another limit is that no data were collected using sleep laboratory methods^5^. Taking their limits into account, data collected in secondary anoneiric subjects represent a minor argument supporting conjecture #2.

## Discussion

Considering the methodological limits of the reviewed studies, we note that there is no major argument against Freud's theory of the basic function of dreaming. By contrast, this theory is corroborated by research results regarding sleep disturbance in brain-damaged patients with anoneira, DRF in neurotic patients, and by the psychophysiological studies in the sleep laboratory.

It must be stressed, however, that the results of these psychophysiological studies suggest that only NREM sleep dreaming may protect sleep against external arousing stimulation. The question still remains whether or not dreaming during REM sleep has a sleep-guarding effect against arousal from internal sources. Indeed, REM sleep entails diffuse brain arousal, particularly pronounced in limbic structures (Dang-Vu et al., 2010), caused by cholinergic discharges originating from the brainstem (Fernández-Mendoza et al., 2009). These ascending arousals probably activate the SEEKING system (Perogamvros and Schwartz, 2012), and this way could trigger drive demands during REM sleep. Thus, REM sleep seems to represent by itself a particular state of psychic arousal, which could explain its high association with dreaming according to Freud's theory^6^.

As an outcome of this review, we conclude that future research on testing Freud's hypothesis of the primary function of dreaming should: (1) focus on sleep laboratory methods; (2) always distinguish REM and NREM sleep dreaming; (3) investigate DRF in conditions (neuropsychiatric, pharmacological) entailing overactivity of the emotional SEEKING system; (4) investigate sleep continuity in primary anoneira, in a well-controlled manner; and (5) investigate sleep continuity in anoneira following posterior brain lesions, in a well-controlled manner.

To conclude, Freud's theory of the basic function of dreaming is empirically testable; it thus display falsifiability and can be considered as a valuable contribution to scientific knowledge, according to the Popperian framework (Popper, 1963). We showed that this aspect of Freud's dream model is partly corroborated by available studies, and could be investigated in more details with appropriated research. Moreover, other aspects of the model, like the wish-fullfilment hypothesis, could also be systematically investigated. Progress in psychoanalysis needs such an approach, which is also the pathway for it takes its rightful place in the field of science.

## Footnotes

^1^Human sleep stages belong to two basic categories (Kryger et al., 2011):

(i) *NREM sleep*: it begins at sleep-onset and is divided into three successive stages (NREM sleep stages 1, 2, and 3), which correspond to a progressive and global slowing of the brain's electrical activity. NREM sleep stage 2 predominates among sleep stages, representing approximately half of total sleep time.
(ii) *REM sleep*: it displays high cortical activation, reminiscent of waking (which led to term it “paradoxical sleep”; Jouvet, 1962).

NREM and REM sleep alternate during the night, resulting in successive sleep cycles (Kryger et al., 2011). Sleep laboratory studies showed that REM sleep is associated with dreaming in approximately 80% of times on average; and NREM sleep stage 2 in approximately 20% of times (see Solms, 2000), but these measures vary with time of the night and gender and age of subjects (see Ruby, 2011).

^2^And being partly disguised by the action of the censor-ego, which retains during sleep part of his capabilities (Freud, 1917/1953).

^3^However, the treatment used was mainly antidepressant medication, which has been found to be associated with decreased DRF, on the contrary (Pace-Schott et al., 2001).

^4^This predicts that patients of the second type would keep dreaming activity in reaction to external and systemic/neuro-peripheral factors.

^5^A corrected statistic for the selection bias could not be obtained from the author. However, a study of DRF in anoneiric patients with the posterior lesion type only is currently in process, which will furthermore provide results from the sleep laboratory (Mark Solms, pers. communication).

^6^This seems corroborated by the fact that enhanced dreaming during REM sleep has been shown to be associated with the progressive cortical activation during nighttime sleep (Antrobus et al., 1995).
